# Edible, Active and Intelligent Food Packaging Polymeric Materials

**DOI:** 10.3390/polym16162251

**Published:** 2024-08-08

**Authors:** Urška Vrabič-Brodnjak

**Affiliations:** Department of Textiles, Graphic Arts and Design, Faculty of Natural Sciences and Engineering, University of Ljubljana, Snežniška 5, 1000 Ljubljana, Slovenia; urska.vrabic@ntf.uni-lj.si; Tel.: +386-1-200-32-82

**Keywords:** bio-based materials, food packaging, innovation, nanotechnology, circularity

## Abstract

With a focus on sustainability and functionality, this Special Issue looks at various topics, including novel food-packaging solutions, bio-based adhesives, and the integration of nanotechnology to improve performance. It emphasizes the importance of understanding limitations and overcoming them through innovation, highlighting the crucial role of bio-based materials in ensuring food safety and quality, especially in the context of the COVID-19 pandemic. Future research areas emphasize the need for holistic approaches that prioritize circularity and sustainability throughout the packaging lifecycle. By fostering collaboration and innovation, this Special Issue aims to make progress towards a more sustainable and resilient future for food packaging.

In the midst of the current global challenges, the discourse on the development of materials, particularly in the field of bio-based materials, has attracted a great deal of attention [1,2,3,4]. The need for sustainable solutions is becoming increasingly apparent, especially in industries that are central to human well-being, such as food packaging [5,6]. With the increasing demand for eco-friendly alternatives, it is imperative to look into the intricacies of bio-based materials and their potential to redefine the landscape of food packaging. Namely, the emergence of bio-based materials offers a promising way to overcome the limitations posed by synthetic counterparts; however, although they are available on the market, several limitations hinder their widespread use. This Special Issue seeks to shed light on these limitations and find ways to overcome them through groundbreaking research and innovation.

One focus of this Special Issue is novel food packaging, which is a critical component in ensuring food safety and quality. The growing trend towards sustainable packaging solutions emphasizes the need to develop materials that not only meet strict quality standards, but also adhere to the principles of environmental sustainability. Against the backdrop of the COVID-19 pandemic, the demand for sustainable packaging solutions has increased significantly, further emphasizing the need for innovative approaches in this area [7,8,9].

Bio-based adhesives are another key aspect that deserve attention. As society strives to transition to a more sustainable future, the development of bio-based adhesives holds great promise for improving the eco-friendliness of packaging materials [10]. By exploring advances in processing techniques, polymer compositions, and characterization methods, researchers can unlock new opportunities in this area, paving the way for greater sustainability and functionality. In addition, the convergence of nanotechnology and coatings offers a wealth of opportunities with which to improve the performance of food packaging. From enhancing barrier properties to integrating active functions, nanotechnology has the potential to revolutionize the food-packaging paradigm and ensure both safety and sustainability [11,12].

With future research efforts in mind, it is essential to emphasize the importance of holistic approaches that encompass the entire life cycles of packaging materials. From the sourcing of raw materials to disposal at the end of life cycles, the principles of the circular economy and sustainability must take center stage. Research into innovative solutions that facilitate the seamless integration of bio-based materials into existing supply chains is essential to realize their full potential.

This Special Issue is an excellent platform for researchers and practitioners to engage with the latest advances in bio-based food-packaging materials. By exploring topics ranging from bio-based adhesives to nanotechnological coatings, we aim to promote a nuanced understanding of the challenges and opportunities in this dynamically evolving field.
